# Preparation of Monoclonal Antibodies Against Porcine Circovirus Type 2 Capsid Protein and Development of a Blocking ELISA for Detection of the Antibody Against the Virus

**DOI:** 10.3390/vetsci13070617

**Published:** 2026-06-25

**Authors:** Haifeng Sun, Qingqing Liu, Shuyan Zhai, Biyue Wu, Zicheng Ma, Yangyang Sun, Kaiyuan Ye, Haoyuan Wang, Yanni Gao, Xianwei Wang, Juan Bai, Ping Jiang

**Affiliations:** 1 Key Laboratory of Animal Disease Diagnostics and Immunology, Ministry of Agriculture, MOE International Joint Collaborative Research Laboratory for Animal Health & Food Safety, College of Veterinary Medicine, Nanjing Agricultural University, Nanjing 210095, China; shf@njau.edu.cn (H.S.); 2022107071@stu.njau.edu.cn (Q.L.); andy-zhai@outlook.com (S.Z.); 13936302480@139.com (B.W.); t2023092@njau.edu.cn (Z.M.); t2025105@njau.edu.cn (Y.S.); 2023807143@stu.njau.edu.cn (K.Y.); why02102@163.com (H.W.); yngao@njau.edu.cn (Y.G.); xwwang@njau.edu.cn (X.W.); 2 Jiangsu Co-Innovation Center for the Prevention and Control of Important Animal Infectious Diseases and Zoonoses, Yangzhou University, Yangzhou 225009, China

**Keywords:** porcine circovirus type 2 (PCV2), blocking ELISA (bELISA), cap protein, monoclonal antibody

## Abstract

Porcine circovirus type 2 (PCV2) is the primary causative agent of a spectrum of porcine circovirus-associated diseases (PCVDs) and remains a major threat to the global swine industry. In this study, ten monoclonal antibodies (mAbs) targeting the Cap protein of PCV2 were generated and characterized. One mAb, designated 4C4, which exhibited high reactivity, strong neutralizing activity, and superior blocking efficacy, was selected for horseradish peroxidase (HRP) labeling. After optimizing the reaction parameters, a blocking ELISA was developed for the detection of the anti-PCV2 antibody. Using receiver operating characteristic (ROC) curve analysis, a cutoff value of 40% was established to distinguish positive from negative serum samples. The sensitivity and specificity of this blocking ELISA method were 98.66% and 100%, respectively. No cross-reactivity was observed with serum antibodies against classical swine fever virus (CSFV), porcine epidemic diarrhea virus (PEDV), porcine deltacoronavirus (PDCoV), porcine reproductive and respiratory syndrome virus (PRRSV), or pseudorabies virus (PRV). Intra-assay and inter-assay repeatability tests yielded coefficients of variation (CVs) all below 10%, confirming the assay’s excellent reproducibility. Simultaneous testing of 312 clinical porcine serum samples using the developed bELISA and a commercial indirect ELISA kit revealed an overall coincidence rate of 99.04%. In addition, the percentage inhibition (PI) in the bELISA was strongly correlated with serum anti-PCV2 neutralizing antibody titers. In conclusion, the blocking ELISA developed herein demonstrates high sensitivity, strong specificity, and good reproducibility, serving as a potentially effective tool for the detection of the anti-PCV2 antibody and epidemiological investigation.

## Simple Summary

Porcine circovirus type 2 (PCV2) severely damages the global pig breeding industry. In this study, 10 monoclonal antibodies (mAbs) targeting the PCV2 Cap protein were prepared and characterized, among which mAb 4C4 with excellent reactivity, neutralizing and blocking activity was selected for HRP labeling. Using the recombinant Cap protein as the coating antigen, a blocking ELISA (bELISA) was optimized and established. The cutoff value was set at 40% via ROC analysis, with 98.66% sensitivity and 100% specificity. No cross-reaction with other common swine pathogens was found, and the assay repeatability was favorable. The detection of 312 clinical sera showed a high coincidence with a commercial kit, and the inhibition rate closely correlated with serum neutralizing antibody titers. This bELISA is reliable for PCV2 antibody detection and epidemiological surveillance.

## 1. Introduction

Porcine circovirus type 2 (PCV2) imposes a substantial economic burden on the global swine industry [1], as the primary causative agent of a spectrum of porcine circovirus-associated diseases (PCVDs) [1,2,3]. Clinical manifestations, including Postweaning Multisystemic Wasting Syndrome (PMWS), Porcine Dermatitis and Nephropathy Syndrome (PDNS), and reproductive failure, severely compromise herd health by inducing immunosuppression, growth retardation, and elevated mortality [2,3,4,5]. Consequently, PCV2 remains a persistent threat to the economic sustainability and profitability of global swine production systems.

To date, at least eight distinct PCV2 genotypes (2a–2h) have been characterized globally. PCV2a, PCV2b and PCV2d are considered the major genotypes, with PCV2d currently being the most prevalent in global pig farms. PCV-2e was identified in swine samples from the USA and Mexico [6].

The development of accurate, rapid, and reliable diagnostic methodologies is a prerequisite for the effective prevention and control of PCV2 [7]. Currently, ELISAs represent the predominant modality in commercial kits for PCV2 antibody detection [8,9,10]. Among these formats, the bELISA has emerged as a preferred method due to its high specificity [11]. By relying on competitive binding between serum antibodies and a specific mAb, this format effectively mitigates non-specific binding, thereby minimizing the false-positive results inherent to complex clinical samples [12,13]. Additionally, the bELISA offers operational simplicity, high throughput, and quantitative analysis capabilities, attributes that are critical for large-scale epidemiological surveillance, vaccine efficacy assessment, and herd immunity monitoring.

The PCV2 capsid (Cap) protein constitutes the primary structural component of the virion [14,15,16], playing an essential role in capsid assembly and serving as the principal immunodominant antigen [17,18]. Given its critical function in inducing neutralizing antibodies and its widespread use in vaccine development [19,20,21], the Cap protein represents an ideal antigen for serological assay development.

mAbs play an irreplaceable role in viral antigenic epitope analysis, pathogen detection, investigation of immune mechanisms, and the development of diagnostic reagents due to their high specificity, strong affinity, good homogeneity, and feasibility for standardized preparation [22,23]. In PCV2 research, neutralizing mAb-based blocking ELISA has become an important tool for antibody monitoring and vaccine efficacy evaluation [12,23,24]. With the continuous evolution of prevalent PCV2 strains, there is an increasing demand for high-quality mAbs with broad-spectrum recognition and stable properties. Accordingly, further enrichment and screening of high-performance mAbs targeting domestic prevalent PCV2 strains, combined with epitope identification and genotype specificity verification, are of great importance for improving the accurate diagnosis and comprehensive prevention of PCV2-associated diseases.

To develop a specific and reliable bELISA for PCV2 antibody detection, ten mAbs targeting the PCV2 Cap protein were generated and systematically characterized. And one mAb, 4C4, with superior performance was selected and labeled with horseradish peroxidase (HRP). Using a recombinant Cap (rCap) protein expressed in a baculovirus expression system (derived from the PCV2b SH strain, GenBank: AY686763.1) as the coating antigen, a bELISA was established and comprehensively evaluated for its sensitivity, specificity, repeatability, cross-reactivity, and correlation with neutralizing antibodies. Furthermore, the established bELISA was validated using IFA as the gold standard reference and compared with a commercial indirect ELISA (iELISA) kit. The results indicated that the bELISA has high sensitivity and specificity for detecting antibodies against PCV2, which can reflect serum neutralizing antibody levels and provide a robust technical platform for the prevention and control of PCV2-associated disease.

## 2. Materials and Methods

### 2.1. Expression and Purification of Recombinant PCV2 Cap Protein

When the density of Sf9 suspension cells reached 2–3 × 10^6^/mL, recombinant baculovirus was inoculated at 0.01–0.1 MOI with serum-free medium, followed by culture at 27–28 °C for 96–120 h. The cell–virus culture medium was harvested when 75% of the cells exhibited cytopathic effects (CPE), and stored at 2–8 °C or frozen at −20 °C for later use. For purification, the harvested virus culture was centrifuged at 5000–6000 r/min at 2–8 °C for 20–30 min to remove cell debris and collect the protein-containing supernatant. The recombinant Cap protein was then purified by cation exchange chromatography using an AKTA protein purification system, and stored at –80 °C.

The purified PCV2 Cap protein was analyzed and confirmed by SDS-PAGE and Western blot with the anti-PCV2 Cap protein mAb 3E5 (made in our lab).

### 2.2. Preparation of Monoclonal Antibodies Against PCV2 Cap Protein

Hybridoma technology was applied for the preparation of anti-Cap mAbs as previously described [25]. Briefly, the purified Cap protein was emulsified with an equal volume of incomplete Freund’s adjuvant (Sigma-Aldrich (Shanghai) Trading Co., Ltd., Shanghai, China) for antigen preparation. Six-week-old female BALB/c mice were subcutaneously immunized with 50 µg/mouse of the prepared antigen 3 times with 3-week intervals between each immunization. The spleen cells of the immunized mice were collected at seven days post-last immunization and fused with SP2/0 myeloma cells using PEG4000 (Sigma−Aldrich (Shanghai) Trading Co. Ltd., Shanghai, China). The confluent cells were cultured in HAT selection medium for seven days before the cell supernatants were screened for anti-Cap antibodies by an indirect enzyme-linked immunosorbent assay (ELISA). Positive cells were subcloned into single-cell clones by limiting dilution, and the subcloning was repeated until all single-cell cloning wells tested positive via indirect ELISA, yielding the final anti-Cap mAbs.

### 2.3. Preparation of Monoclonal Antibody Ascites

Six- to eight-week-old healthy BALB/c mice received an intraperitoneal injection of 0.5 mL of sterile liquid paraffin. Seven days later, 1–2 × 10^6^ hybridoma cells were intraperitoneally inoculated into each mouse. The hybridoma cells proliferated abundantly as ascitic tumors in the peritoneal cavity. After 7–10 days, the mice exhibited markedly distended and fluctuant abdomens, lethargy and ruffled fur, indicating that the ascites could be harvested. The collected ascites was transferred to centrifuge tubes and centrifuged at 4000 rpm for 10 min. The clear intermediate layer was collected and stored at −80 °C for further use.

### 2.4. SDS-PAGE and Western Blot

The protein samples were respectively denatured with 5× loading buffer (Beyotime Biotech Co., Ltd., Shanghai, China) at 100 °C for 10 min, and then loaded (20 µL per sample) onto 12% SDS-PAGE gels. The Bio-Rad Mini-Protean Tetra system was used for electrophoresis at 80 V for 2 h. For Coomassie blue staining analysis, the SDS-PAGE gels were stained with the staining solution for 30 min, and then decolored with the decolorization solution before imaging. For Western blot analysis, the proteins on the SDS-PAGE gel were then transferred onto nitrocellulose filter membranes and blocked with 5% (*w*/*v*) non-fat dry milk/tris-buffered saline for 4 h at room temperature. After washing with PBST, the membranes were incubated with the primary mAb for 2 h at room temperature. Following another five washes, secondary antibodies of HRP-labeled goat anti-mouse IgG were added for a 1 h incubation at room temperature, and the blots were scanned and analyzed with a digital imaging system (Beyotime Biotech Co., Ltd., Shanghai, China) after a final wash step.

### 2.5. Immunofluorescence Assay (IFA)

For the reactivity analysis between the prepared mAbs and the PCV2 Cap protein, the confluent monolayers of PK-15 cells infected with the PCV2 SH strain were washed with PBST (Phosphate-Buffered Saline Tween-20) (Vazyme Biotech Co., Ltd., Nanjing, China) 3 times. They were then fixed with 80% acetone pre-cooled at −20 °C. After washing, the 10 hybridoma ascites of anti-PCV2 Cap protein monoclonal antibodies (MAbs) diluted 1:128 were added to quadruplicate wells. Rabbit anti-PCV2 polyclonal serum (1:128) was used as the positive control, and anti-PRRSV N protein MAb (1:128) served as the negative control. The plates were incubated at 37 °C for 1 h, and washed three times with PBST. 488-conjugated goat anti-mouse IgG or 488-conjugated goat anti-rabbit IgG was added, followed by incubation at 37 °C for 30 min. After a final PBST wash step, the cells exhibiting fluorescence signals were observed under a fluorescence microscope(Carl Zeiss AG, Oberkochen, Germany).

### 2.6. Virus Neutralization Test (VNT)

A VNT combined with an IFA was employed to detect the neutralizing activity of the 10 mAbs, as well as to analyze 118 clinical pig sera for ROC analysis and an additional 40 clinical serum samples. Both mAb ascites and clinical sera were serially two-fold diluted with serum-free DMEM to obtain a series of dilutions from 1:8 to 1:1024, mixed with an equal volume of the PCV2 SH strain (200 TCID_50_), and incubated at 37 °C for 1 h as described previously [26]. The mixtures were added to the wells of a 96-well plate containing confluent PK-15 cells (100 μL/well), and cultured for 72 h at 37 °C with 5% CO_2_. Meanwhile, PCV2 (200 TCID_50_) mixed with an equal volume of specific pathogen-free (SPF) pig serum (1:8, made in our lab) was used as positive control, and DMEM alone was used as the negative control. Following PBST washing and acetone fixation as described above, the IFA was performed to determine the neutralizing effect of the mAbs and clinical sera, using a rabbit anti-PCV2 polyclonal serum (1:1000) and 488-conjugated goat anti-rabbit IgG. After a final PBST wash step, the cells exhibiting fluorescence signals were observed under a fluorescence microscope(Carl Zeiss AG, Oberkochen, Germany). At least three microscopic fields were used to capture microscopic images. The microscopic images were analyzed to count the fluorescence-positive cells. The neutralizing antibody titer was defined as the highest dilution showing more than 50% inhibition of fluorescence-positive cells compared to the PCV2 positive control as described previously [26]. A neutralizing antibody titer less than 1:8 was considered negative, while a titer equal to or greater than 1:8 was considered positive.

### 2.7. Isotype Determination of the Anti-Cap mAbs

The isotypes of the 10 anti-Cap mAbs were determined using a Mouse Monoclonal Antibody Subtype Identification Kit (Cat No.: PK20003), (Proteintech, Wuhan, China) according to the instructions.

### 2.8. Conservation Analysis of the Linear B-Cell Epitope on PCV2 Cap Protein Recognized by mAb 4C4

The B-cell antigen epitope recognized by the 4C4 monoclonal antibody was previously identified in our laboratory [27]. The Cap protein amino acid sequences from 25 representative strains covering 8 gene subtypes of PCV2 (PCV2a~PCV2h) were downloaded from the GenBank database. All sequences were imported into BioEdit software (version 7.1.9, North Carolina State University, Raleigh, NC, USA) for sequence alignment to evaluate the conservation of the identified antigen epitope among different PCV2 genotypes.

### 2.9. PCV Species Type Specificity Identification

To get recombinant Cap proteins of different PCV species, the ORF2 sequences of PCV1 (GenBank: FJ475129.2), PCV2 (GenBank: AY686763.1), PCV3 (GenBank: OR059205.1) and PCV4 (GenBank: MT311854.1) were synthesized and cloned into the pcDNA3.1(+) expression vector fused with a Flag tag by the GenScript Biotech Corporation ( Nanjing, China). The theoretical molecular weights of the four rCap proteins are 25.7 kDa, 25.8 kDa, 23.7 kDa, and 25.2 kDa, respectively. No linker and signal peptide sequences were designed in any of the recombinant expression constructs. A total of 2 μg of each recombinant plasmid was transfected into HEK293T cells. Cells were harvested and lysed at 24 h post-transfection. The expression of the recombinant proteins was verified by Western blot using an anti-Flag antibody as the primary antibody. The diluted ascites of the 4C4 monoclonal antibody was then used as the primary antibody in a Western blot to identify the recognition specificity of the 4C4 mAb against different PCV species.

### 2.10. Preparation of Enzyme-Labeled Antibodies

Eight-week-old BALB/c mice were employed to generate the mAb ascites using the 4C4 hybridoma cell line. The ascites was purified by Zhongding Biotechnology Co., Ltd. (Nanjing, China) followed by HRP labeling at GenScript Biotechnology Co., Ltd. (Nanjing, China). The purity was analyzed by sodium dodecyl sulfate-polyacrylamide gel electrophoresis (SDS-PAGE), and the titer was determined by ELISA to select the optimal working concentration.

### 2.11. bELISA

The purified PCV2 Cap protein was diluted in carbonate buffer (pH 9.6) to the optimal concentration, and 100 μL was added to each well of a 96-well ELISA plate. The plate was coated at 37 °C for 2 h, then washed three times with phosphate-buffered saline containing 0.05% Tween-20 (PBST, pH 7.2) for 5 min each. After blocking with 200 μL of 1% BSA in PBST at 37 °C for 3 h, the plate was washed as described above. Test serum diluted in PBST (1:5) was added (100 μL/well) and incubated at 37 °C for 1 h, followed by washing. The HRP-conjugated mAb 4C4 diluted in PBST (1:2000) was added (100 μL/well) and incubated at 37 °C for 1 h, then the plate was washed again. For the screening of the mAbs with blocking activity, the mAb diluted in PBST (1:2000) was added (100 μL/well) and incubated at 37 °C for 1 h. After washing, the HRP-conjugated anti-mouse IgG diluted in PBST (1:3000) was added (100 μL/well) and incubated at 37 °C for 1 h, then the plate was washed again. TMB substrate solution (100 μL/well) was added for color development at 37 °C for 15 min. The reaction was terminated by adding 50 μL of 2 mol/L sulfuric acid per well, and the absorbance at 450 nm (OD_450nm_) was measured using a microplate reader. The percentage inhibition (PI) was calculated using the formula: PI = [(OD_450nm_ of negative control serum − OD_450nm_ of test serum)/OD_450nm_ of negative control serum] × 100%.

#### 2.11.1. Optimization of the Conditions of bELISA

##### Selection of Optimal Antigen Coating Concentration and Serum Dilution

A checkerboard titration was performed to determine the optimal coating concentration of the Cap protein and the dilution of the test serum. The antigen and PCV2 standard positive/negative sera were serially diluted, and each dilution was tested in duplicate. The PI of the positive serum was calculated, and the conditions yielding the highest PI were selected as optimal.

##### Selection of Antigen Coating Temperature and Reaction Time

Three coating conditions were compared: 4 °C for 16–24 h, 37 °C for 2 h, and 37 °C for 2 h followed by 4 °C for 16–24 h. The optimal antigen coating concentration was used, and the PI of the positive serum was measured to select the best temperature and time.

##### Selection of Optimal Blocking Buffer and Reaction Time

Four blocking buffers were evaluated: 2% gelatin, 1% BSA, 5% non-fat milk, and 10% non-fat milk (all diluted in PBST). After selecting the optimal blocking buffer, blocking times of 1 h, 2 h, and 3 h at 37 °C were compared to determine the best condition.

##### Selection of Optimal Incubation Time for the Serum Samples

The serum samples at the optimal dilution were incubated at 37 °C for 0.5 h, 1 h, 1.5 h, or 2 h. The PI of the positive serum was calculated to select the optimal incubation time.

##### Selection of Optimal Working Concentration of HRP-Conjugated mAb and Incubation Time

Based on the titer determined by direct ELISA, the HRP-conjugated mAb was serially diluted in PBST. The PI of the positive serum was measured to select the optimal working concentration. Then, the HRP-conjugated mAb at the optimal concentration was incubated at 37 °C for 0.5 h, 1 h, or 1.5 h. The PI of the positive serum was calculated to select the optimal incubation time.

##### Selection of Optimal Reaction Time of TMB Substrate

After incubating the HRP-conjugated mAb under optimal conditions, the TMB substrate was added and incubated at 37 °C for 5 min, 10 min, 15 min, or 20 min. The OD_450nm_ values were measured, and the PI of the positive serum was calculated to determine the optimal color development time.

### 2.12. Cutoff Value, Diagnostic Sensitivity and Specificity of bELISA

The cutoff value of the bELISA method with optimal diagnostic sensitivity and specificity was determined using 63 PCV2-positive and 55 PCV2-negative pig serum samples, which were confirmed by IFA. GraphPad Prism 8.0 (San Diego, CA, USA) was utilized to analyze the receiver operating characteristic (ROC) curves, as well as the diagnostic sensitivity and specificity of the bELISA. Diagnostic sensitivity and specificity were calculated as: Sensitivity = TP/(TP + FN) × 100%. Specificity = TN/(TN + FP) × 100%. TP, true positive; FP, false positive; TN, true negative; FN, false negative.

### 2.13. Cross-Reactivity of bELISA

To evaluate the analytical specificity of the bELISA assay, 5 porcine serum samples containing antibodies against classical swine fever virus (CSFV) (provided by Dr. Bin Zhou at Nanjing Agricultural University), porcine epidemic diarrhea virus (PEDV), porcine deltacoronavirus (PDCOV), porcine reproductive and respiratory syndrome virus (PRRSV), or pseudorabies virus (PRV) strains (made in our lab), were detected by the bELISA. The specific antibodies were previously confirmed using commercial ELISA kits (IDEXX, Westbrook, ME, USA).

### 2.14. Assessment of the bELISA Repeatability

In order to assess the repeatability of the bELISA method, four positive samples and two negative samples were tested. Intra-assay and inter-assay repeatability were evaluated using three batches of ELISA plates coated with rCap proteins purified 3 times. The mean PI value and the coefficient of variation (CV) were calculated based on three replications of each test.

### 2.15. Correlation Analysis of Antibody Levels Measured by bELISA and Neutralizing Activity

Based on the PI values of the sera detected with the bELISA, forty serum samples were selected and categorized into four groups each with 10 samples: negative (PI < 40%), weakly positive (40% ≤ PI < 60%), moderately positive (60% ≤ PI < 80%), and strongly positive (PI ≥ 80%). The neutralizing activity of the serum samples was detected with VNA, and they were classified into four grades based on the neutralizing titer (NT): negative (−) (<1:8), low (+) (1:8 ≤ NT < 1:32), moderate (++) (1:32 ≤ NT < 1:128), and high (+++) (≥1:128). Spearman correlation analysis was carried out between bELISA PI values and the corresponding neutralizing activity grades to explore the correlation between the bELISA results and the actual serum neutralizing capacity.

### 2.16. Comparative Test

The established bELISA and a commercial indirect ELISA (iELISA) for anti-PCV2 antibody detection kit were used to test 312 clinical serum samples, aiming to compare and evaluate the reliability of the bELISA method.

### 2.17. Statistical Analysis

GraphPad Prism 8.0 software (GraphPad Software, Inc., San Diego, CA, USA) was used for the statistical analyses. The data are expressed as the means ± SDs. The intra- and inter-assay variations were evaluated by the CV.

### 2.18. Mice

BALB/c mice were purchased from the Animal Experiment Center of Yangzhou University. These mice were used for immunization, the preparation of hybridoma cells, and the production of monoclonal antibody ascites.

### 2.19. Cell Lines

HEK293T and SP2/0 cell lines were obtained from the Key Laboratory of Animal Disease Diagnostic and Immunology, College of Veterinary Medicine, Nanjing Agricultural University (Nanjing, China), and maintained in our laboratory.

### 2.20. Viruses

The PCV2 SH strain (GenBank: AY686763.1) was originally obtained from the Key Laboratory of Animal Disease Diagnostic and Immunology, College of Veterinary Medicine, Nanjing Agricultural University, Nanjing, China, and has been stably preserved and maintained in our laboratory. This strain was used for the construction of recombinant PCV2 Cap plasmids, as well as subsequent IFA, VNT and Western blot assays.

### 2.21. Antibodies

The anti-PCV2 polyclonal antibody serum (rabbit) and mAb 3E5 ascites (mouse) were originally obtained from the Key Laboratory of Animal Disease Diagnostic and Immunology, College of Veterinary Medicine, Nanjing Agricultural University, Nanjing, China, and have been maintained in our laboratory. They were all identified to have good reactivity with PCV2.

## 3. Results

### 3.1. Generation and Identification of the Anti-Cap mAbs

**Figure 1:**
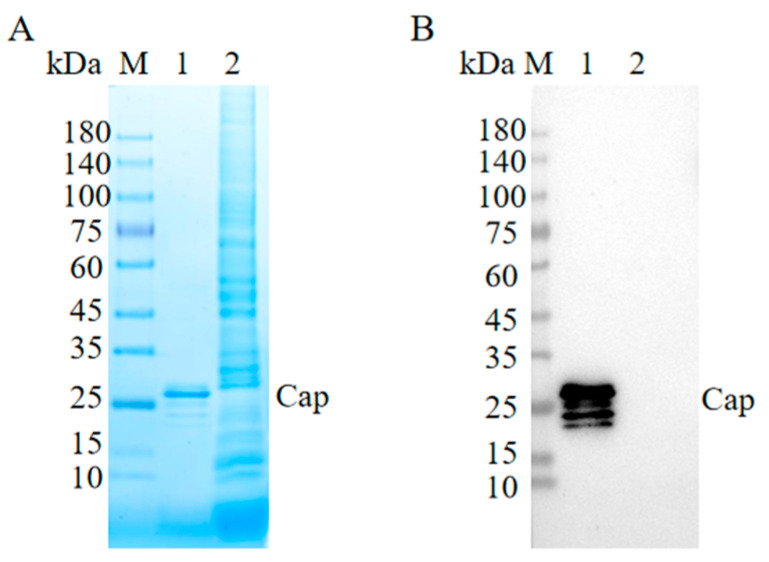
Identification of rCap protein with SDS-PAGE (**A**) and Western blot using Anti-PCV2 Cap protein mAb 3E5 (**B**). M: Marker; Lane 1: purified rCap protein, Lane2: Negative control (SF9 cells).

The rCap of PCV2 was expressed and purified from the SF9 cells infected with the recombinant baculovirus containing PCV2 ORF2. SDS-PAGE and Western blot revealed that the rCap protein was 28 kDa in size, and it could react with the anti-PCV2 Cap mAb 3E5 (prepared in our lab) (Figure 1). After three rounds of immunization, ELISA revealed that the titer of antigen-specific antibodies in the mouse serum reached 1:102,800. This was sufficient for hybridoma fusion and subsequent experiments.

After fusion between the mouse spleen cells and SP2/0 cells, the hybridoma cell lines secreting the antibodies against the PCV2 Cap protein, named 2B6, 3D4, 3E8, 3E6, 3F6, 4C4, 5A6, 5G4, 5H7, 6A7, were selected by ELISA. After 15 passages, the titers of the mAbs detected by ELISA in cell cultures were 1: 6400~1:409,600. The titers of the mAbs in ascites were 1:1.02 × 10^5^~1:1.31 × 10^7^ (Table 1).

**Figure 2:**
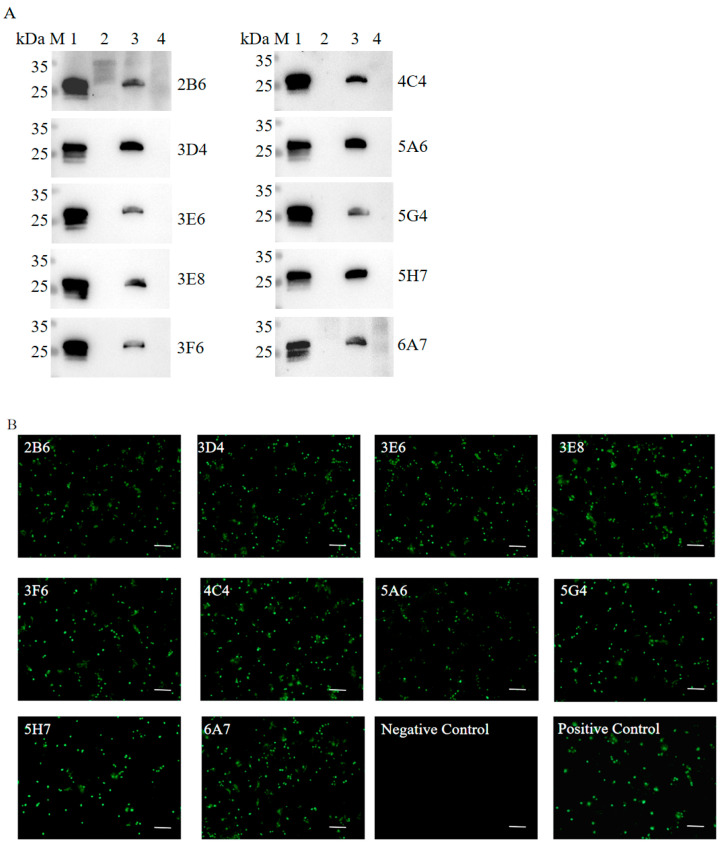
(**A**) Western blot analysis of mAbs reactivity against PCV2 and rCap protein. M: Marker; Lane 1. Cap recombinant protein; Lane 2. SF9 cell control; Lane 3. PCV2; Lane 4. PK-15 cell control. (**B**) IFA of mAbs reactivity against PCV2 infected PK-15 cells. Positive control: anti-PCV2 Cap ployclonal antibody; Negative control:anti-PRRSV N protein MAb ascites. The white horizontal lines in each image panel correspond to scale bars of 100 μm.

The specificity of the mAbs was verified by Western blot with the recombinant Cap protein and PCV2-infected PK15 cells, using 2B6, 3D4, 3E8, 3E6, 3F6, 4C4, 5A6, 5G4, 5H7, and 6A7 as primary antibodies, respectively. As shown in Figure 2A, the 10 mAbs all showed good reactivity and specificity against the two forms of the Cap protein. Meanwhile, IFA results showed that all the 10 mAbs could specifically bind to PCV2-infected PK-15 cells, with clear fluorescence signals observed. No specific fluorescence was detected in the negative control (Figure 2B). This indicates that all the 10 mAbs have good reactive properties with the PCV2 Cap protein.

### 3.2. Neutralizing Activity of the mAbs

**Figure 3:**
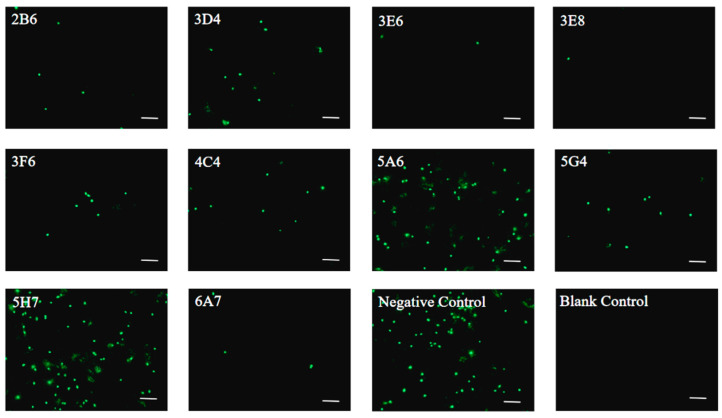
Neutralizing activity of the mAbs (dilution of 1:8) against PCV2 detected by IFA. Negative control: anti-PRRSV N protein mAb; Blank control: PK-15 cells.The white horizontal lines in each image panel correspond to scale bars of 100 μm.

A VNT combined with Ian FA was employed to detect the neutralizing activity of the 10 mAbs. The results demonstrated that 8 out of the 10 mAbs (2B6, 3D4, 3E8, 3E6,3F6, 4C4, 5G4, 6A7) exhibited strong neutralizing activity (Table 1), with a significant reduction in the number of fluorescence-positive cells compared with the negative control. The remaining two mAbs (5A6, 5H7) showed no obvious neutralizing activity, and the number of fluorescence-positive cells was not significantly different from that of the negative control (Figure 3).

### 3.3. Isotype Determination of the mAbs

The results of the monoclonal antibody isotyping are shown in Table 2. The heavy chain isotype was IgG2a for mAbs 2B6, 3D4, 3E8, 3E6, 3F6, 4C4, 5A6 and 5G4, IgG2b for mAb 6A7, and IgG1 for mAb 5H7. All mAbs possessed the kappa light chain.

### 3.4. Screening of the mAb Used in bELISA and Its Labeling with HRP

**Figure 4:**
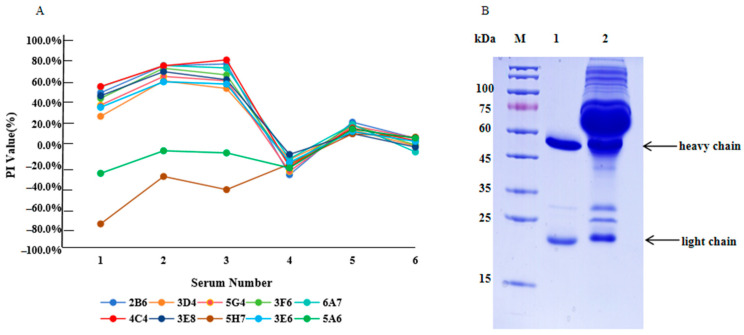
Screening and identification of mAb 4C4 labeled with HRP. (**A**) Blocking activity of the 10 mAbs in bELISA. (**B**) SDS-PAGE analysis of purified mAb 4C4. M: Marker, Lane 1: Purified mAb 4C4, Lane 2: Unpurified mAb 4C4.

To screen the mAbs for use in bELISA, the blocking effect of the mAbs was detected + using bELISA. Results showed that all eight mAbs with neutralizing activity exhibited blocking effects, while no blocking effect was detected in the other two mAbs without neutralizing activity. And the 4C4 had the characteristics of good reactivity, strong neutralizing activity and the optimal blocking effect (Figure 4A). Thus, 4C4 was selected for purification and labeling with HRP, which was used in the blocking ELISA.

SDS-PAGE analysis of the purified 4C4 revealed distinct bands corresponding to the heavy and light chain bands (Figure 4B). The concentration of the purified mAb was determined to be 0.507 mg/mL, while the titer of the HRP-conjugated mAb reached 1:25,600.

### 3.5. High Conservation of the mAb 4C4 Epitope Among Diverse PCV2 Genotypes Except PCV2e

**Figure 5:**
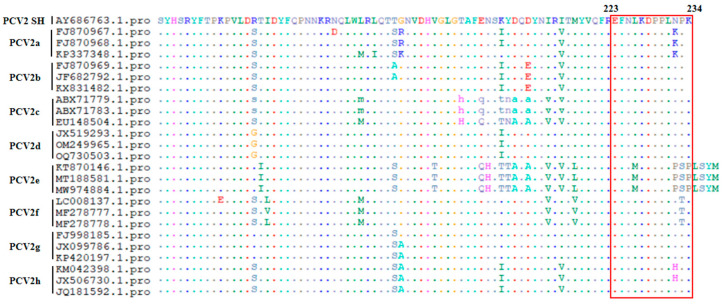
Conservation of the mAb 4C4 epitope among diverse PCV2 genotypes. Red box indicates residues 223–234 of PCV2 Cap protein.

The epitope recognized by mAb 4C4 was previously identified in our laboratory [27]. Sequence analysis of the Cap proteins from 25 PCV2 strains belonging to different genotypes was performed using BioEdit 7.1.9 software (Figure 5). The epitope 223EFNLKDPPLNPK234 exhibited high conservation among the different genotypes. PCV2a, PCV2f and PCV2h had only one amino acid mutation site compared to PCV2b and PCV2d. PCV2e had four amino acids mutation sites in the epitope compared to other PCV2 genotypes.

### 3.6. Species Specificity of mAb 4C4 for PCV2

**Figure 6:**
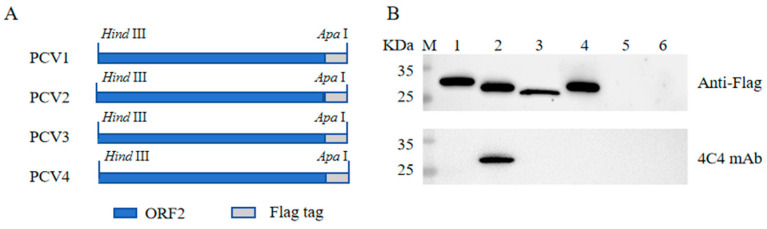
Recombinant plasmid construction and genotype specificity analysis of mAb 4C4. (**A**) Linear schematic of PCV1–PCV4 ORF2-Flag inserts. Each full-length ORF2 fused with Flag tag at C-terminal was inserted at *Hind*III/*Apa*I of pcDNA3.1(+) vector. (**B**) The recombinant Cap proteins were detected by Western blot using anti-Flag antibody and mAb 4C4. M: Marker; Lanes 1–4: recombinant Cap proteins of PCV1, PCV2, PCV3 and PCV4, respectively; Lane 5: Empty vector control; Lane 6: Mock control.

The PCV species-specific reactivity of mAb 4C4 was assessed by Western blot against the recombinant Cap proteins of PCV1, PCV2, PCV3, and PCV4. As shown in Figure 6, all four recombinant Cap proteins expressed in HEK293T cells could be detected with the anti-Flag antibody, with molecular weights of approximately 30 kDa, 28 kDa, 26 kDa and 28 kDa, respectively, while no signal was observed in the empty vector and mock control groups. The mAb 4C4 only reacted specifically with the PCV2 Cap protein but not with the PCV1, PCV3, or PCV4 Cap proteins, indicating that mAb 4C4 is PCV2 species-specific.

### 3.7. Optimization of the Reacting Conditions of bELISA

Through checkerboard titration with three anti-PCV2 negative and three anti-PCV2 positive serum samples, the optimal coating antigen concentration was 0.75 μg/mL, with an optimal HRP-4C4 dilution of 1:2000. Furthermore, a serum dilution of 1:5 was selected as optimal, as this condition yielded the maximum percentage inhibition (PI) values (Table 3).

### 3.8. Cut-Off Value, Sensitivity and Specificity of the bELISA

**Figure 7:**
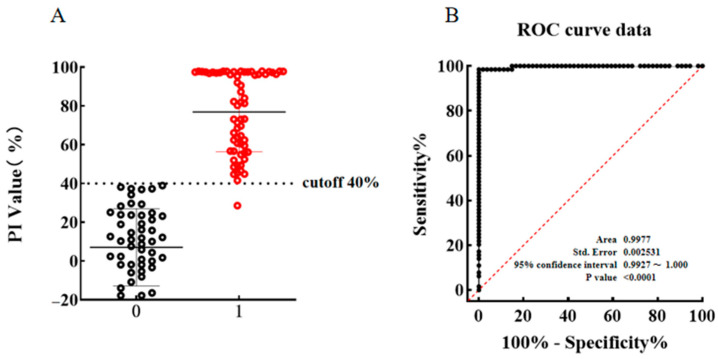
ROC analysis for cutoff value determination of positive and negative samples. (**A**) bELISA interactive dot plot analysis. The ordinate denotes PI of serum samples, and the abscissa denotes bELISA test results (0 = negative; 1 = positive). (**B**) ROC curve analysis of the bELISA.

To establish the diagnostic cutoff value, a total of 54 anti-PCV2 negative and 64 anti-PCV2 positive serum samples were evaluated by bELISA using the optimized condition. ROC curve analysis demonstrated high diagnostic accuracy, yielding an Area Under the Curve (AUC) of 0.9977 (standard error = 0.002531, 95% confidence interval: 0.9927~1.000, *p* < 0.0001) (Figure 7B). Based on the maximal Youden’s index, a cutoff value of 40% was selected. At this threshold, the bELISA exhibited 98.66% diagnostic sensitivity and 100% specificity (Figure 7A). Consequently, the interpretation criteria were defined as follows: samples with a PI ≥ 40% are considered positive, while samples with a PI < 40% are classified as negative.

### 3.9. Cross-Reactivity of the bELISA

**Figure 8:**
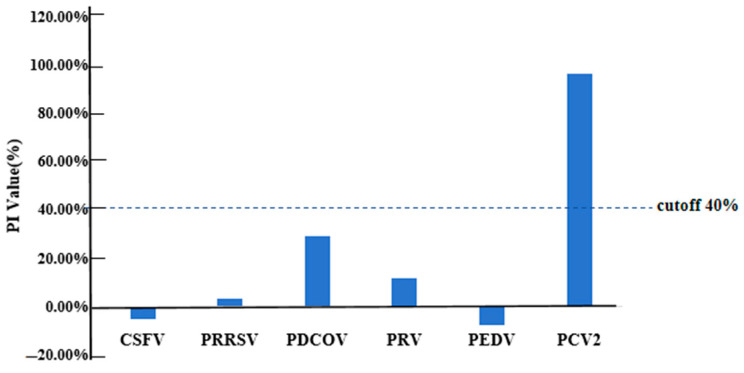
Verification of cross-reactivity of the blocking ELISA (bELISA).

To evaluate the cross-reactivity, the assay was tested with anti-CSFV, PRRSV, PDCoV, PRV, and PEDV positive sera. As shown in Figure 8, all heterologous sera had PI values < 40%, while the anti-PCV2 positive serum had a high PI value of 96.4%. This clear distinction indicates no cross-reactivity of this method with antibodies against these major swine viral pathogens.

### 3.10. Repeatability Test

To evaluate the repeatability of the bELISA, four anti-PCV2 positive and two negative serum samples were tested. The intra-assay coefficients of variation (CVs), calculated from replicates within the same batch, ranged from 0.36% to 7.55%. Whereas, the inter-assay CVs, determined across three independent batches, ranged from 1.46% to 7.58% (Table 4). All calculated CVs remained below the 10% acceptance threshold, demonstrating that the established bELISA possesses excellent repeatability.

### 3.11. The Results of Comparative Test

To evaluate the clinical performance and concordance between the bELISA and iELISA, a total of 312 serum samples were tested by both methods. The coincidence rate between the two methods was 99.04%, and the Kappa value was 0.977 (Table 5). This confirms the high specificity and favorable accuracy of the bELISA in PCV2 antibody detection.

### 3.12. Correlation Analysis of Antibody Levels Measured by bELISA and VNT

**Figure 9:**
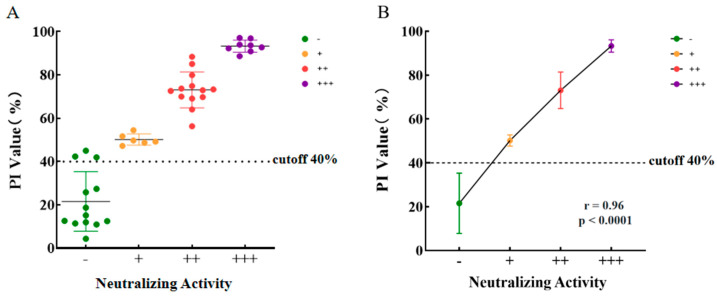
Correlation analysis between bELISA and VNT. (**A**) Distribution of PI values with different neutralizing activity of the serum. (**B**) Correlation between PI values and neutralizing activity. Neutralizing activity: −: <1:8 (negative); +: 1:8 ≤ NT < 1:32 (weak); ++: 1:32 ≤ NT < 1:128 (moderate); +++: ≥1:128 (strong).

To evaluate the correlation between the bELISA and the neutralizing activity, forty serum samples categorized by bELISA were evaluated with the VNT. As shown in Figure 9, the PI category corresponded to the NT in each group: PI < 40% to negative neutralizing activity (<1:8, −), 40% ≤ PI < 60% to weak activity (1:8 ≤ NT < 1:32, +), 60% ≤ PI < 80% to moderate activity (1:32 ≤ NT < 1:128, ++), and PI ≥ 80% to strong activity (≥1:128, +++). Spearman correlation analysis confirmed a significant positive correlation between the bELISA PI values and the neutralizing activity grades (r = 0.96, *p* < 0.0001, Figure 9B). It demonstrates that this bELISA can serve as a reliable tool for detecting the serum neutralizing antibody levels against PCV2.

## 4. Discussion

Serological surveillance is critical for the prevention and control of PCV2 [28,29], as accurate and reliable antibody detection underpins vaccine efficacy evaluation, epidemiological investigations, and disease control strategies [30,31]. Currently, several serological techniques for PCV2 are widely used, each with distinct advantages and limitations [32,33,34]. The indirect ELISA remains the predominant format in commercial kits, favored for its cost-effectiveness, high-throughput potential, and operational simplicity. However, its dependence on secondary antibody binding often leads to non-specific reactions, resulting in false-positive results, especially when testing complex clinical samples derived from herds with co-infections [33]. IFA can detect early antibody responses but requires specialized fluorescence microscopy, skilled operators for result interpretation, and strict sample handling conditions, thereby limiting its applicability for on-site or large-scale epidemiological investigations [35]. Although the colloidal gold immunochromatographic assay (GICA) provides rapid [36], on-site detection with minimal technical requirements, its low sensitivity and inability to generate quantitative data limit its utility for precise immune status assessment and vaccine efficacy monitoring. A bELISA based on specific mAbs could avoid these drawbacks by competitive binding, which reduces non-specific interference and improves specificity [12,13]. Meanwhile, the bELISA enables quantitative detection via percentage inhibition (PI), making it more suitable for high-throughput screening, immune monitoring, and vaccine efficacy evaluation [9]. In this study, we prepared 10 mAbs against the PCV2 Cap protein and established a bELISA using the superior mAb 4C4, providing a reliable tool for detecting anti-PCV2 antibodies.

The success of a bELISA largely depends on the selection of a suitable coating antigen and a highly specific detection antibody. A high-performance bELISA relies heavily on a high-quality antigen and specific monoclonal antibody. The PCV2 Cap protein was chosen as the coating antigen due to its unique biological properties: as the major structural protein of PCV2, it is highly conserved among different PCV2 strains, contains key epitopes for host immune recognition, and serves as the primary target for neutralizing antibody production [37,38,39]. In this study, the recombinant Cap protein expressed via the baculovirus system (PCV2b SH strain, GenBank: AY686763.1) was verified to have high purity and correct immunological activity through SDS-PAGE and Western blot analysis. The minor bands below 28 kDa are attributed to incomplete glycosylation modifications rather than protein degradation or impurities [40], which do not affect the purity and immunological activity of the protein. Meanwhile, systematic identification of 10 mAbs against PCV2 showed that the obtained hybridoma cell lines exhibited favorable genetic stability and potent antibody-secreting capability, suitable for long-term ascites production. Subtyping revealed that the 10 mAbs were predominantly of the IgG2a isotype, with all light chains being of the kappa type. Western blot and IFA confirmed that all 10 mAbs specifically bound to the recombinant Cap protein and PCV2-infected cells, but showed no reactivity with uninfected PK-15 cells or SF9 cells, demonstrating excellent specificity and no cross-reactivity for PCV2 detection. The VNT indicated that eight mAbs displayed distinct virus-neutralizing activity, while the remaining two mAbs (5A6, 5H7) had no neutralizing activity. Blocking ELISA results showed that all eight neutralizing mAbs possessed blocking activity, while the other two non-neutralizing mAbs had no blocking effect. Among the 10 anti-Cap mAbs characterized by IFA, VNT and bELISA, mAb 4C4 exhibited the strongest reactivity, neutralizing activity, and blocking efficiency. The linear B-cell epitope recognized by mAb 4C4 was accurately identified as ^223^EFNLKDPPLNPK^234^ [27]. Sequence alignment and conservation analysis further demonstrated that this epitope is highly conserved among the globally dominant and prevalent strains, PCV2b and PCV2d. The high conservation of the target epitope ensures that the bELISA could maintain stable reactivity against most prevalent PCV2 field strains, laying a foundation for broad-spectrum detection in diverse epidemiological investigations.

Systematic optimization of reaction conditions is critical to maximizing the sensitivity, specificity, and repeatability of a bELISA. In this study, a checkerboard titration was used to determine the optimal coating concentration of rCap (0.75 μg/mL) and the dilution of HRP-conjugated mAb 4C4 (1:2000), ensuring the optimal balance between signal intensity and assay sensitivity. The coating condition of 37 °C for 2 h followed by 4 °C for 16–24 h was selected to enhance antigen adsorption onto the ELISA plate, while 1% BSA was identified as the optimal blocking buffer to minimize non-specific binding without affecting specific antigen–antibody interactions. Incubation times for the test serum (1 h) and the HRP-conjugated mAb 4C4 (1 h) were optimized to achieve sufficient binding while maintaining assay efficiency, and a TMB reaction time of 15 min was chosen to ensure clear signal differentiation between positive and negative samples. These optimized conditions were selected according to “the highest PI value”, although without statistical optimization such as a multivariate analysis of variance or the response surface methodology, which may ignore the interaction between factors and affect the robustness of the method.

To examine the cutoff value of bELISA, ROC curve analysis was performed with 118 serum samples confirmed by IFA, a gold-standard reference method. Thus, a cutoff value of 40% PI was established, with a diagnostic sensitivity of 98.44% and a specificity of 100%. The high specificity effectively eliminated false-positive results commonly observed in the indirect ELISA, which is particularly important for complex clinical samples. The method showed no cross-reactivity with anti-CSFV, PEDV, PDCoV, PRRSV and PRV positive sera. In addition, the mAbs 4C4 could not recognize the recombinant Cap proteins of PCV1, PCV3, and PCV4, confirming the ability of this bELISA to specifically detect PCV2 antibodies under multi-infection conditions. The intra-assay and inter-assay coefficients of variation were all below 10%, indicating excellent repeatability. Spearman correlation analysis revealed a significant positive correlation (r = 0.96, *p* < 0.0001) between the bELISA PI values and serum neutralizing antibody titers, indicating that the bELISA can reliably reflect the anti-PCV2 neutralizing antibody levels.

Nevertheless, this study has some limitations that need to be addressed in the future. The antigen epitope recognized by 4C4 is conserved in the dominant and prevalent genotypes PCV2b and PCV2d. However, PCV2e has four amino acid mutation sites in the epitope compared to other genotypes. Here, no experiments were performed to verify whether mAb 4C4 is capable of binding the epitope of PCV2e. This might pose a possible risk of affecting antibody binding and decreasing the sensitivity of the bELISA. Since our laboratory does not have the serum antibodies against PCV1, PCV3 and PCV4, we lacked a direct specificity verification of the cross-reactivity of the bELISA with the serum antibodies against PCV. As an alternative, we performed a Western blot to characterize the genotype-specific binding profiles of mAb 4C4 to PCV1, PCV2, PCV3 and PCV4 Cap proteins, which indirectly reflects the genotype specificity of the bELISA. The cross-reactivity of the bELISA with the serum antibodies against PCV1, PCV2, PCV3 and PCV4 needs to be detected in the future. In addition, the current bELISA cannot differentiate virus-infected from vaccinated animals (DIVA), which limits its application in distinguishing natural infection from vaccine-induced immunity.

In conclusion, in this study, ten anti-PCV2 Cap mAbs were generated and identified. Among them, mAb 4C4 has good reactivity, strong neutralizing activity, an optimal blocking effect and strict PCV2 species type specificity. Subsequently, a bELISA was established for detecting the anti-PCV2 antibody, having high sensitivity, strong specificity, good repeatability, and a significant correlation with neutralizing antibodies. It provides a valuable tool for virus antibody detection, vaccine efficacy evaluation, and epidemiological surveillance, thereby supporting the effective prevention and control of PCVDs.

## Figures and Tables

**Table 1:** The titer of the mAbs in hybridoma culture supernatant and mouse ascites detected with indirect ELISA and VNT.

mAbs Name	ELISA (Generation)			ELISA	NT
	P5	P10	P15	Ascites	Ascites
2B6	1:256,000	1:204,800	1:409,600	1:1.64 × 10^6^	1:512
3D4	1:12,800	1:25,600	1:12,800	1:1.64 × 10^6^	1:256
3E8	1:12,800	1:12,800	1:12,800	1:3.28 × 10^6^	1:256
3E6	1:1600	1:1600	1:1600	1:8.19 × 10^5^	1:128
3F6	1:25,600	1:409,600	1:409,600	1:6.55 × 10^6^	1:256
4C4	1:102,400	1:102,400	1:204,800	1:6.55 × 10^6^	1:512
5A6	1:6400	1:6400	1:6400	1:8.19 × 10^5^	<1:8
5G4	1:12,800	1:25,600	1:25,600	1:1.31 × 10^7^	1:512
5H7	1:3200	1:6400	1:6400	1:8.19 × 10^5^	<1:8
6A7	1:12,800	1:12,800	1:25,600	1:1.02 × 10^5^	1:128

**Table 2:** Identification results of the mAbs subtypes.

Monoclonal Antibody	Subclass and Types (OD_450nm_)							
	IgG1	IgG2a	IgG2b	IgG3	IgGA	IgGM	Kappa	Lambda
2B6	0.209	2.491	0.152	0.073	0.065	0.109	1.8	0.079
3D4	0.504	2.439	0.442	0.1	0.068	0.087	1.531	0.114
3E8	0.24	2.227	0.289	0.071	0.054	0.071	1.329	0.073
3E6	0.675	2.184	0.222	0.076	0.066	0.224	1.351	0.097
3F6	0.154	2.254	0.233	0.065	0.056	0.086	1.316	0.083
4C4	0.225	1.825	0.233	0.064	0.055	0.112	1.078	0.069
5A6	0.226	1.676	0.407	0.104	0.063	0.234	1.331	0.084
5G4	0.151	1.542	0.283	0.073	0.056	0.082	0.969	0.069
5H7	2.316	0.472	0.291	0.064	0.054	0.055	1.049	0.068
6A7	0.103	0.454	2.679	0.075	0.055	0.1	1.875	0.076

**Table 3:** Optimization of the reaction conditions of bELISA for anti-PCV2 antibody.

Optimized Conditions	Antigen Coating	Blocking Conditions	Serum to Be Tested	HRP Labeled 4C4	TMB Reaction Time
Concentration/dilution	0.75 μg/mL	1% BSA	1:5	1:2000	
Reaction conditions	37 °C 2 h followed by 4 °C 16–24 h	37 °C 3 h	37 °C 1 h	37 °C 1 h	37 °C 15 min

**Table 4:** Intra- and inter-assay repeatability of the mAb 4C4-based bELISA.

Samples	Intra-Assay			Inter-Assay		
	Mean PI (%)	SD	CV	Mean PI (%)	SD	CV
Positive 1	82.52	0.023	2.74%	80.82	0.042	5.20%
Positive 2	69.34	0.030	4.30%	68.71	0.031	4.53%
Positive 3	55.67	0.011	1.98%	53.28	0.020	3.70%
Positive 4	42.48	0.010	2.45%	43.16	0.022	5.17%
Negative 1	26.29	0.009	3.55%	25.07	0.010	3.91%
Negative 2	16.55	0.008	4.89%	15.73	0.007	4.55%

**Table 5:** Comparisons of the bELISA and iELISA by detecting field serum samples.

		bELISA			
	Serum Samples	Positive Sample No.	Negative Sample No.	Total	Coincidence
iELISA	Positive sample no.	221	3	224	99.04%
	Negative sample no.	0	88	88	
	Total	221	91	312	
Kappa Value		0.977			

## Data Availability

All data is contained within the article or Supplementary Material. The original contributions presented in this study are included in the article/Supplementary Material. Further inquiries can be directed to the corresponding author.

## Supplementary Materials

The following supporting information can be downloaded at: https://www.mdpi.com/article/10.3390/vetsci13070617/s1, File S1: The original image of WB in the figure.
