# Supplementary figures and images for: Preparation of Monoclonal Antibodies Against Porcine Circovirus Type 2 Capsid Protein and Development of a Blocking ELISA for Detection of the Antibody Against the Virus
Source: Vet Sci. 2026 Jun 25;13(7):617. doi: 10.3390/vetsci13070617 (PMC13417405; doi:10.3390/vetsci13070617)

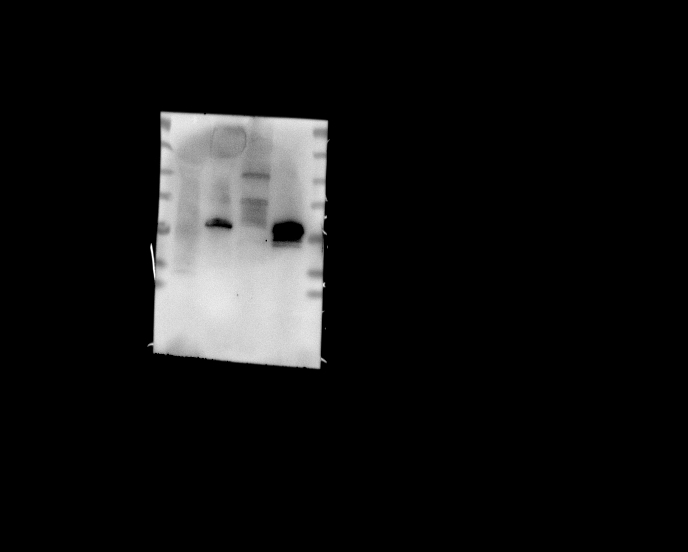

Supplement: Supplementary file 1 [file vetsci-13-00617-s001.zip › File S1/2B6.jpg]

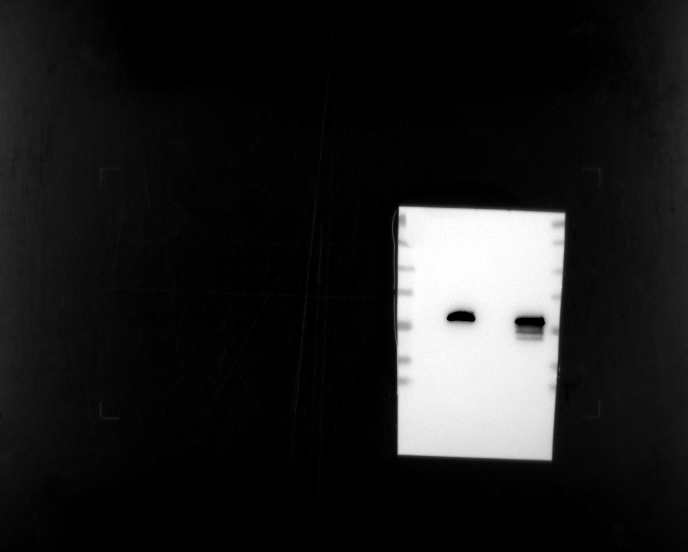

Supplement: Supplementary file 1 [file vetsci-13-00617-s001.zip › File S1/3D4.jpg]

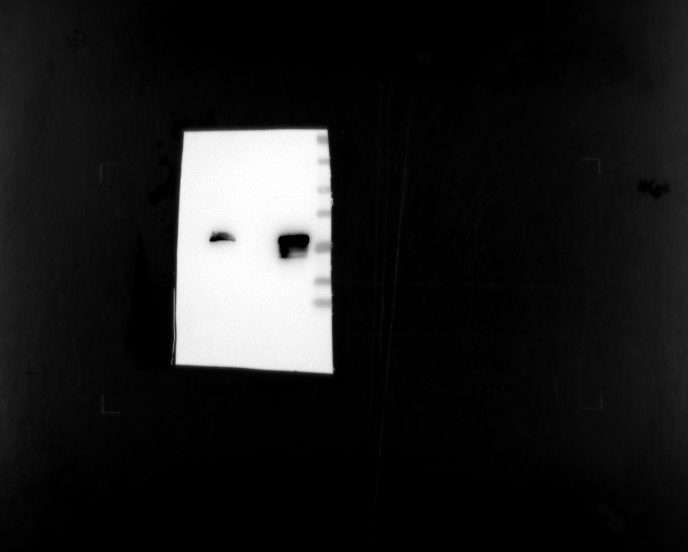

Supplement: Supplementary file 1 [file vetsci-13-00617-s001.zip › File S1/3E5.jpg]

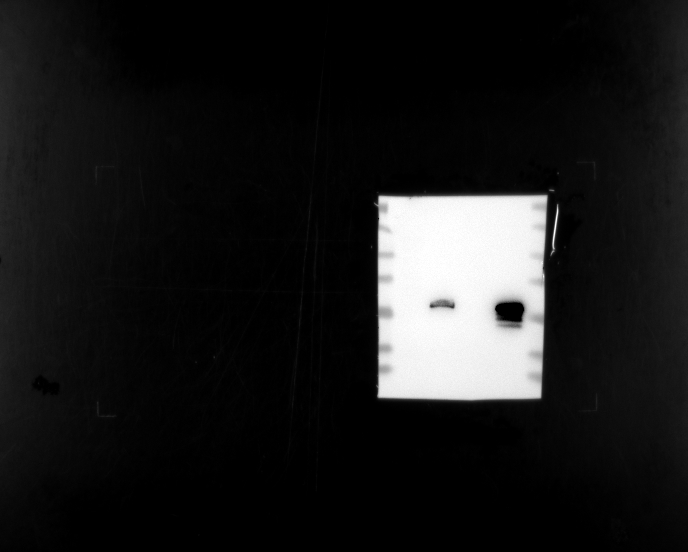

Supplement: Supplementary file 1 [file vetsci-13-00617-s001.zip › File S1/3E6.jpg]

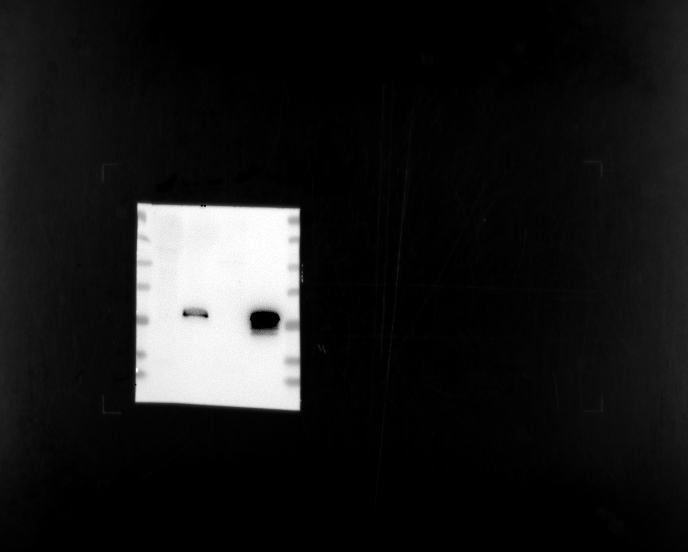

Supplement: Supplementary file 1 [file vetsci-13-00617-s001.zip › File S1/3F6.jpg]

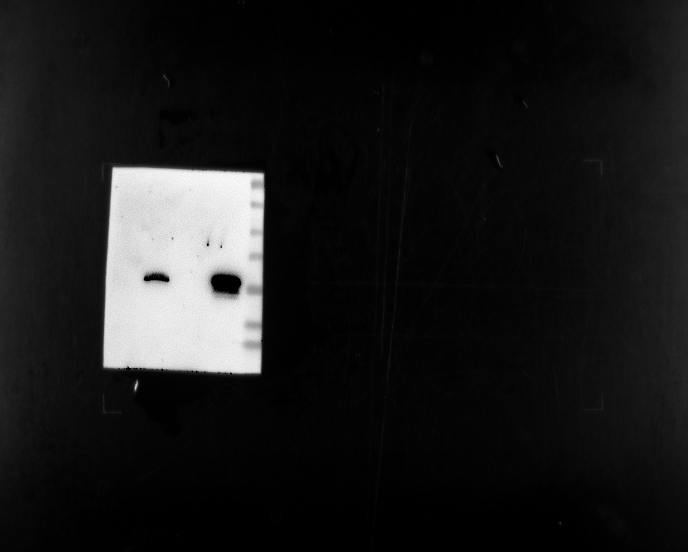

Supplement: Supplementary file 1 [file vetsci-13-00617-s001.zip › File S1/4C4.jpg]

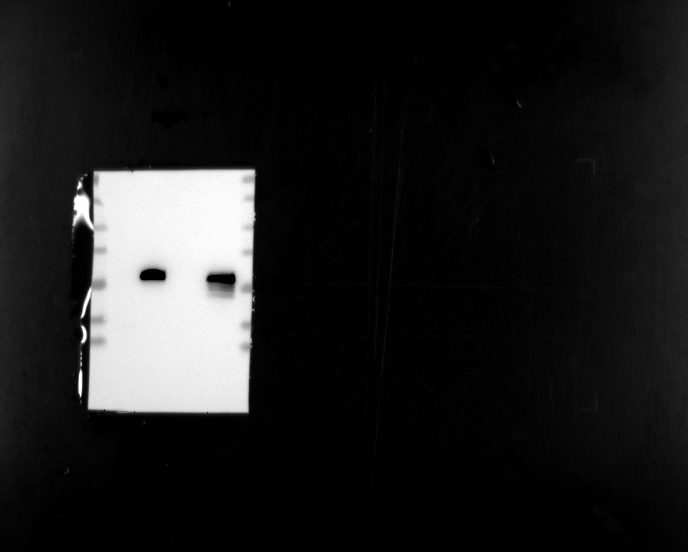

Supplement: Supplementary file 1 [file vetsci-13-00617-s001.zip › File S1/5A6.jpg]

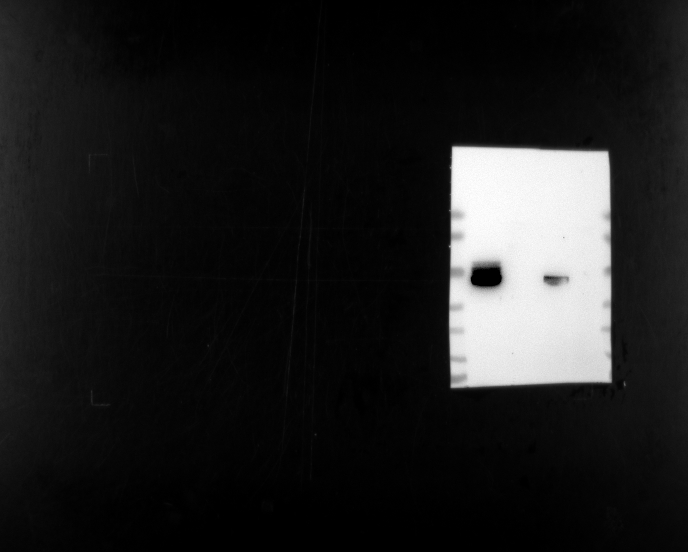

Supplement: Supplementary file 1 [file vetsci-13-00617-s001.zip › File S1/5G4.jpg]

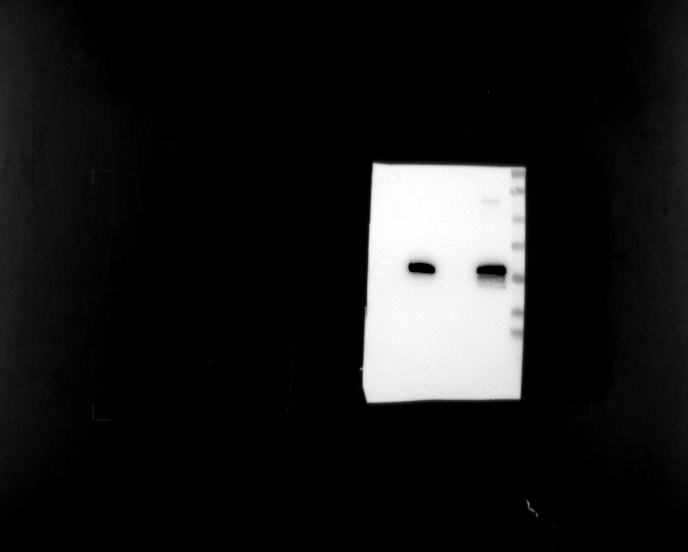

Supplement: Supplementary file 1 [file vetsci-13-00617-s001.zip › File S1/5H7.jpg]

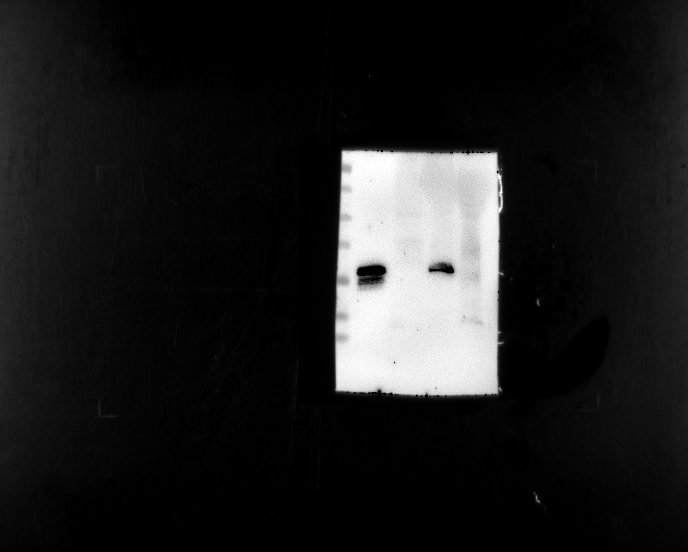

Supplement: Supplementary file 1 [file vetsci-13-00617-s001.zip › File S1/6A7.jpg]

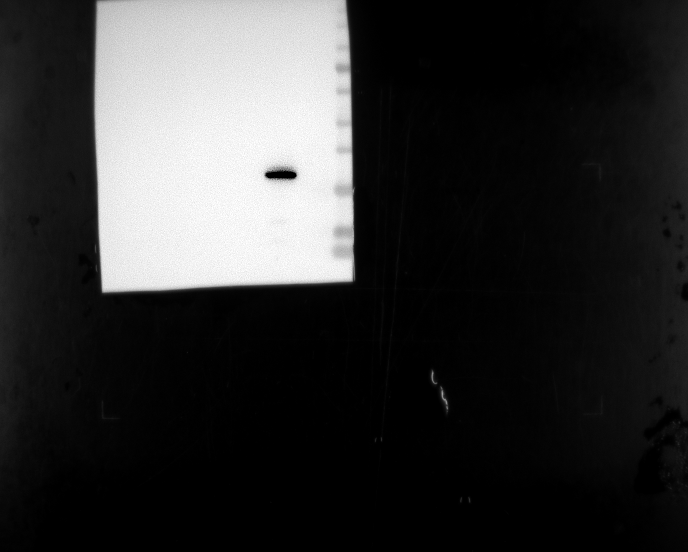

Supplement: Supplementary file 1 [file vetsci-13-00617-s001.zip › File S2/Anti-4C4.tif]

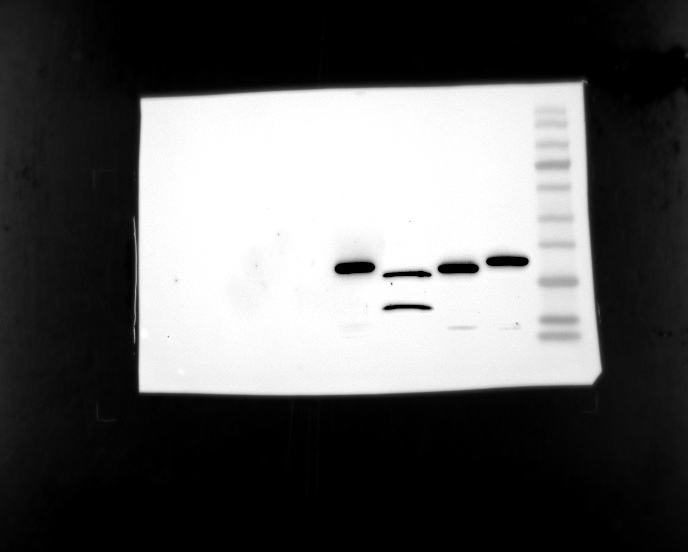

Supplement: Supplementary file 1 [file vetsci-13-00617-s001.zip › File S2/Anti-Flag.tif]

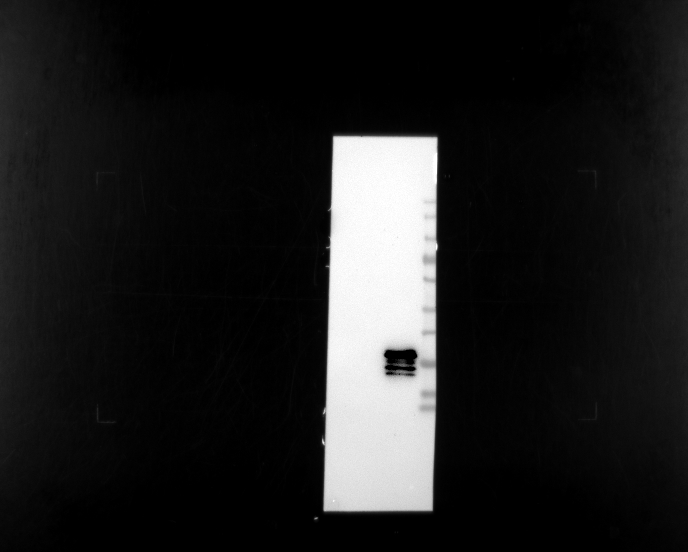

Supplement: Supplementary file 1 [file vetsci-13-00617-s001.zip › Figure S1.jpg]
